# Relative contributions of hydraulic dysfunction and carbohydrate depletion during tree mortality caused by drought

**DOI:** 10.1093/aobpla/plx069

**Published:** 2017-12-08

**Authors:** Yongxin Dai, Lin Wang, Xianchong Wan

**Affiliations:** 1 Institute of New Forestry Technology, Chinese Academy of Forestry, Beijing, P.R. China; 2 College of Forestry, Shanxi Agricultural University, Taigu, Shanxi, P.R. China

**Keywords:** Carbon limitation, drought-induced mortality mechanism, drought modes, hydraulic failure, *P. orientalis*, *R. pseudoacacia*

## Abstract

Drought-induced tree mortality has been observed worldwide. Nevertheless, the physiological mechanisms underlying this phenomenon are still being debated. Potted *Robinia pseudoacacia* and *Platycladus orientalis* saplings were subjected to drought and their hydraulic failure and carbon starvation responses were studied. They underwent simulated fast drought (FD) and slow drought (SD) until death. The dynamics of their growth, photosynthesis, water relations and carbohydrate concentration were measured. The results showed that during drought, growth and photosynthesis of all saplings were significantly reduced in both species. The predawn water potential in both species was ~ −8 MPa at mortality. The percentage loss of conductivity (PLC) was at a maximum at mortality under both FD and SD. For *R. pseudoacacia* and *P. orientalis*, they were >95 and ~45 %, respectively. At complete defoliation, the PLC of *R. pseudoacacia* was ~90 % but the trees continued to survive for around 46 days. The non-structural carbohydrate (NSC) concentrations in the stems and roots of both FD and SD *R. pseudoacacia* declined to a very low level near death. In contrast, the NSC concentrations in the needles, stems and roots of *P. orientalis* at mortality under FD did not significantly differ from those of the control, whereas the NSC concentrations in SD *P. orientalis* stems and roots at death were significantly lower than those of the control. These results suggest that the duration of the drought affected NSC at mortality in *P*. *orientalis.* In addition, the differences in NSC between FD and SD *P*. *orientalis* did not alter mortality thresholds associated with hydraulic failure. The drought-induced death of *R. pseudoacacia* occurred at 95 % PLC for both FD and SD, indicating that hydraulic failure played an important role in mortality. Nevertheless, the consistent decline in NSC in *R. pseudoacacia* saplings following drought-induced defoliation may have also contributed to its mortality.

## Teaser

Different tree species have various strategies in response to different drought types. Black locust (a deciduous tree) and arborvitae (an evergreen tree) present different physiological death processes when exposed to fast− (FD) or slow−drought (SD) stress. In this study, black locust shed its leaves when subjected to drought, underwent hydraulic failure and carbon depletion regardless of FD or SD. However, arborvitae retained its needles when exposed to drought, only suffered hydraulic failure under FD, while it experienced both hydraulic failure and carbon depletion under SD.

## Introduction

In recent years, climate change has caused drought resulting in extensive woodland tree mortality and forest decline worldwide (Anderegg and Anderegg 2013; Bouche *et al.* 2014; Gaylord *et al.* 2015). The causes, consequences, climatic thresholds and models of drought-induced tree mortality have been under close study (Breshears *et al.* 2009; Martínez-Vilalta *et al.* 2012; Anderegg *et al.* 2013; McDowell *et al.* 2013; Gustafson 2014; Mitchell *et al.* 2014; Gu *et al.* 2015). Their underlying physiological mechanisms are still being debated (Anderegg *et al.* 2012; Sevanto *et al.* 2014; Meir *et al.* 2015). Recently, three hypotheses have been proposed concerning the mechanisms of drought-related tree mortality: hydraulic failure, carbon starvation and biotic attack (McDowell *et al.* 2008). Many studies have supported these hypotheses (Galvez *et al.* 2011; Kulac *et al.* 2012; Plaut *et al.* 2012; Barigah *et al.* 2013; Mitchell *et al.* 2013, 2014; Klein *et al.* 2014; Sevanto *et al.* 2014). Two physiological responses to drought are defined as follows (Pangle *et al.* 2015): (i) hydraulic failure—low water potential due to decreased soil water content or increased transpiration rate impeding long-distance water transport and causing cavitation embolism or dehydration; (ii) carbon starvation—stomatal closure and restricted carbon translocation, uptake and assimilation during prolonged drought.

Hydraulic failure is a major cause of woody plant mortality during drought (Choat 2013). Carbon starvation is relatively slow and may occur in the late stages of prolonged drought (McDowell 2011; Kulac *et al.* 2012; Klein *et al.* 2014). Drought alters carbon allocation and partitioning among organs or tissues above- or below-ground. It may reduce non-structural carbohydrate (NSC) concentrations in organs rather than whole plants (McDowell and Sevanto 2010; Galvez *et al.* 2011; Gruber *et al.* 2012). Most studies in this area have focused on *Pinaceae* spp. (Galiano *et al.* 2011; Adams *et al.* 2013; Anderegg and Anderegg 2013; Mitchell *et al.* 2013; Poyatos *et al.* 2013; Sevanto *et al.* 2014; Gaylord *et al.* 2015). Isohydric stomatal regulation in *Pinaceae* spp. makes them susceptible to carbon starvation (McDowell *et al.* 2008) but the evidence for this response is limited (O’Grady and Mitchell 2015). Many tree species shed their leaves when challenged with drought. This strategy reduces transpirational area and protects plant integrity (Galvez *et al.* 2011; Ryan 2011). Near-zero photosynthesis and drought-induced canopy defoliation significantly alter carbon metabolism dynamics (Poyatos *et al.* 2013). To date, however, little research has been conducted on this phenomenon in species that readily defoliate in response to water deficit.

Recently, the interaction between hydraulic failure and carbon starvation has been studied (Anderegg and Anderegg 2013; Nardini *et al.* 2013; Mencuccini 2014; Sevanto *et al.* 2014). The experimental objective was to determine whether hydraulic failure or carbon limitation plays a major role in drought-related tree mortality (Pangle *et al.* 2015). Several studies emphasize that drought intensity and duration significantly influence tree mortality (McDowell *et al.* 2008; Plaut *et al.* 2012; Mitchell *et al.* 2013; Pangle *et al.* 2015). Some studies demonstrated that the major cause of tree death under intense drought is hydraulic failure (Anderegg and Anderegg 2013; Barigah *et al.* 2013; Nardini *et al.* 2013; Urli *et al.* 2013). In most reports, only one species with a single drought response or exposure to one drought type was used to determine the changes in their hydraulic relations or carbohydrate dynamics (Galvez *et al.* 2011; Anderegg and Anderegg 2013; Kukowski *et al.* 2013; Piayda *et al.* 2014). Mitchell *et al.* (2013) demonstrated that drought response strategies determined the relative contributions of hydraulic failure and carbohydrate depletion during mortality. Klein *et al.* (2011) suggested that drought intensity and duration influenced hydraulic function and carbon assimilation in *Pinus halepensis*. Therefore, it is useful to know how trees with various drought management strategies respond to different drought types.

*Robinia pseudoacacia* and *Platycladus orientalis* are used for afforestation in northern China. Recently, drought has caused a severe die-off of *R. pseudoacacia*. In contrast, this mortality has not been observed in *P. orientalis*. *Robinia pseudoacacia* is deciduous and usually drought-resistant. Nevertheless, it is more prone to dehydration during rapid growth and profligate water use than the evergreen *P. orientalis* (Mantovani *et al.* 2014). Unlike *P. orientalis*, *R. pseudoacacia* easily defoliates during drought. Stomatal conductance and photosynthesis are also sensitive to drought stress in *R. pseudoacacia* (Wang *et al.* 2017). *Robinia pseudoacacia* and *P. orientalis* may, therefore, differ in terms of hydraulic failure, carbon starvation and mortality when subjected to drought. The aim of this study was to determine how drought conditions and response strategies affect tree mortality. Potted *R. pseudoacacia* and *P. orientalis* were exposed to simulated intense (fast) and moderate (slow) drought. Growth, water relations, photosynthetic rates and NSC concentrations were observed during drought. Water relations and carbon budget responses under various drought conditions were compared between the two species. We hypothesized that (i) NSC is lower in SD than FD plants at the point of mortality; thus, SD plants tend to suffer carbon depletion while FD plants is more susceptible to hydraulic failure; (ii) death occurs at higher water potentials and/or lower percentage loss of conductivity (PLC) in NSC-depleted plants; and (iii) *R*. *pseudoacacia* is more susceptible to carbon depletion than *P*. *orientalis* because the former easily defoliates under drought stress.

## Methods

### Plant materials and treatments

Three-year-old *R. pseudoacacia* and *P. orientalis* saplings were obtained from a local nursery at Shanxi Agricultural University. They were maintained for 3 months before the experiments. Two hundred saplings of each species were planted in pots (35 cm × 35 cm) filled with a 2:1 volumetric mixture of topsoil and sand. The saplings were regularly watered and received Osmocote^®^ Extra fertilizer (N-P-K: 16-9-12 + 2MgO + TE, Everris International B.V., The Netherlands). On 1 June 2014, saplings of similar size from each species **[see Supporting Information—Table S1]** were selected and randomly divided into three groups of 50 each. The treatments were (i) fast drought (FD), (ii) slow drought (SD) and (iii) control (CK), respectively. All plants were placed in the nursery, grown in open field conditions and covered with plastic cloth overnight and when it rained. The experimental site was located at Taigu (37°26′N, 112°32′E, altitude 780 m), Shanxi Province, northern China. The mean annual rainfall is ~458 mm. Precipitation is at a maximum from June to August and at a minimum from December to February. The mean annual air temperature is 9.8 °C. The mean air temperature is lowest in January (−6.2 °C) and highest in July (23.6 °C).

The control plants were watered to field capacity daily. Soil water content was monitored via a portable soil moisture meter (TRME-T3 TDR, IMKO, Ettlingen, Germany). The FD treatment was achieved by completely withholding irrigation until the plants died. The SD treatment consisted of gradually drying the soil by reducing the water supply to 60 % of the previous irrigation until the amount of water provided to each plant was <10 mL. Eventually, water was no longer given to the plants, and the soil was allowed to dry down until the plants died. The SD experiments were run for 4–5 months, between June and October, until the trees died. Plant death was diagnosed by staining leaf and branch samples with 0.1 % neutral red to ensure that no living cells remained (Sevanto *et al.* 2014). Staining was conducted only after complete defoliation of *R. pseudoacacia* or when *P. orientalis* needles started browning at intervals of ~1 week. For *P. orientalis*, both needles and stems were stained. Both needles and stems were equally effective at indicating mortality.

### Water potential and PLC

Predawn water potential (Ψ_pd_) was measured at 4:30–5:30 a.m. every 2 weeks on five small twigs per treatment for each species using a Scholander-type pressure chamber (PMS1505D-EXP, PMS Instrument Company, Albany, OR, USA). Predawn PLC was also measured on five branches the same day. For all treatments and both species, a branch segment 30–40 cm long was excised under water and then immediately brought to a nearby laboratory. A 3-cm-long segment was cut and trimmed under water from the cut end of the branch using a new razor blade. Percentage loss of conductivity was measured with a low-pressure flow apparatus (Sperry *et al.* 1988; Sun *et al.* 2011). The flow solution consisted of 0.025 mol L^−1^ KCl in degassed distilled water filtered through a 0.22 µm micropore membrane. For *R. pseudoacacia*, initial conductivity (*K*_i_) and maximal conductivity (*K*_max_) were measured before and after a 5-min flush at 0.175 MPa to remove embolisms. For *P. orientalis*, *K*_i_ and *K*_max_ were measured before and after ≥4 h vacuum extraction to remove embolisms (Anderegg and Anderegg 2013). A preliminary experiment showed that for *R. pseudoacacia* there was no significant difference in PLC whether it was flushed by high pressure or vacuum extraction [PLC = (1 − *K*_i_/*K*_max_) × 100 %].

### Net photosynthetic rate and stomatal conductance

The photosynthetic rate and stomatal conductance were measured in mature leaves on a sunny morning (9:00–11:00 a.m.) using the Li-6400XT portable photosynthesis system (LI-COR, Lincoln, NE, USA). The instantaneous photosynthetic rate and stomatal conductance were measured under an active photon flux intensity of 1500 µmol m^−2^ s^−1^ and an ambient CO_2_ concentration of 410 ± 30 μmol mol^−1^ in a red-blue light chamber. The light saturation point for both *R. pseudoacacia* and *P. orientalis* is ≤1500 µmol m^−2^ s^−1^. Readings were taken after gas exchange steady-state was attained. The gas exchange was measured weekly before complete defoliation for *R. pseudoacacia* and every 2 weeks for *P. orientalis*.

### Observations, growth measurements and sample harvest

Leaf colour change and defoliation timing were observed and recorded over the course of the experiment. Increments in sapling basal diameter, height and biomass were measured. Before treatment initiation and after plant death, the basal diameters and the heights of six plants per treatment were measured. Six plants of each species were harvested for biomass and NSC determination at the start of the treatment. Six of the plants from each species and treatment used to determine the biomass increment were also harvested after death. To evaluate NSC, leaves (needles), branches (stems) and roots were sampled every 20 days until plant death. An entire plant was dissected into leaves (needles), branches (stems), main roots, coarse roots and fine roots to determine biomass increments based on dry weight. Portions of the stems and main roots were further separated into phloem and xylem for carbohydrate measurement. Phloem is the bark minus the outermost periderm. Xylem is the entire woody part of the stem. Immediately after collection, all samples were heated in an oven at 110 °C for 30 min to stop any biological activity. They were then dried to a constant weight at 65 °C for 48 h.

### Carbohydrate analyses

The NSC consisted mainly of soluble sugar (SS) and starch (St). Oven-dried materials (needles, stem phloem, stem xylem, root phloem and root xylem) were ground to a fine powder and filtered through a 100-mesh sieve. The SS and St concentrations were measured using a modified anthrone method (Mitchell *et al.* 2013). Soluble sugar was extracted from 0.1 g plant tissue powder with 5 mL 80 % aqueous ethanol (v/v). The mixture was incubated in a water bath at 80 °C for 30 min and then centrifuged at 3500 r.p.m. for 10 min. The supernatant was collected and the residue re-extracted twice as before. The supernatants were pooled and destained with 10 mg active carbon at 80 °C for 30 min. The SS concentration was determined photometrically at 615 nm in the presence of anthrone–sulfuric acid reagent. The residue was hydrolyzed from St to SS (glucose) with perchloric acid solution. Two millilitres of distilled water was added to the residue and the mixture was boiled in a water bath at 100 °C for 15 min. Two millilitres of 9.2 mol L^−1^ perchloric acid was added to the mixture and left to react with it for 15 min. After centrifugation at 4000 r.p.m. for 10 min, the residue was re-extracted with 2 mL of 4.6 mol L^−1^ perchloric acid for another 15 min. The residue was washed twice with 5–6 mL distilled water. The pooled supernatants were used for photometric St determination. Carbohydrate concentration (%) was recorded as the ratio of carbohydrate weight to dry tissue weight.

### Statistical analyses

Data from growth between pre-drought and drought treatments, and parameters at mortality between drought intensities and species were analysed by the *t*-test. Two-way ANOVA was used for testing differences between drought treatments and the control over the experimental time. The variables included hydraulic behaviour, gas exchange, SS, St and NSC concentration for each species (α = 0.05). All tests were performed using SAS v. 8.1 (SAS Institute Inc., Cary, NC, USA). The significance level was set to α = 0.05 in all cases.

## Results

### Plant mortality

For both species, the SD plants survived significantly (*P* < 0.05) longer than the FD plants (Table 1). The FD plants had almost the same mean survival time (*P* = 0.59): 74.90 ± 2.31 days and 75.43 ± 4.92 days for *R. pseudoacacia* and *P. orientalis*, respectively. Nevertheless, SD *P. orientalis* survived for 1 month longer than SD *R. pseudoacacia* (101.97 ± 4.14 days and 132.03 ± 5.51 days for *R. pseudoacacia* and *P. orientalis*, respectively) (Tables 2 and 3). Intraspecific variation in time-to-mortality among individuals was 1–2 weeks. The leaves of FD *R. pseudoacacia* turned yellow and abscised just over 10 days after the treatment started. The saplings of *R. pseudoacacia* were leafless within 1 month but survived for another ~45 days. The leaves of SD *R. pseudoacacia* turned yellow and fell about 1 month after those of the FD plants. In contrast, the leaves of both FD and SD *P. orientalis* remained green until just before death, when they turned brown and withered.

**Table 1.** The statistical significance (*P* value) of the effect of drought intensity and species on studied parameters at mortality of plants. Significant at α = 0.05. RP, *Robinia pseudoacacia*; PO, *Platycladus orientalis*; FD, fast drought; SD, slow drought; Ψ_pd_, predawn water potential; PLC, percentage loss of conductivity; P_n_, photosynthetic rate; g_s_, stomatal conductance; NSC, non-structural carbohydrate; stem-p, stem phloem; stem-x, stem xylem; root-p, root phloem; root-x, root xylem. Bold digits indicate significant differences between drought treatments and control, or between fast drought and slow drought treatments (*P* < 0.05).

Survival	FD	SD	*P* value	Leaf NSC	FD	SD	*P* value
RP	74.9	102.0	**<0.01**	RP	7.22	2.90	**<0.01**
PO	75.4	132.0	**<0.01**	PO	7.49	6.44	0.25
*P* value	0.59	**<0.01**		*P* value	0.77	**<0.01**	
**Ψ_pd_**	**FD**	**SD**	***P* value**	**Stem-p NSC**	**FD**	**SD**	***P* value**
RP	−7.48	−7.36	0.69	RP	1.16	1.34	0.55
PO	−8.44	−8.03	0.23	PO	3.78	1.61	**0.025**
*P* value	**<0.01**	0.07		*P* value	**<0.01**	0.60	
**PLC**	**FD**	**SD**	***P* value**	**Stem-x NSC**	**FD**	**SD**	***P* value**
RP	97.6	96.3	0.30	RP	1.35	1.16	0.19
PO	45.2	43.5	0.44	PO	4.51	3.65	0.33
*P* value	**<0.01**	**<0.01**		*P* value	**<0.01**	**0.013**	
**P_n_**	**FD**	**SD**	***P* value**	**Root-p NSC**	**FD**	**SD**	***P* value**
RP	−0.28	−0.44	**<0.01**	RP	1.38	1.05	0.35
PO	−0.40	−0.67	**<0.01**	PO	6.31	2.03	**<0.01**
*P* value	0.21	**<0.01**		*P* value	**<0.01**	0.10	
**g_s_**	**FD**	**SD**	***P* value**	**Root-x NSC**	**FD**	**SD**	***P* value**
RP	1.84	3.40	0.10	RP	1.72	1.02	0.15
PO	2.44	3.84	0.40	PO	6.53	0.80	**<0.01**
*P* value	0.46	0.77		*P* value	**<0.01**	0.47	

**Table 2.** Growth and survival time of *Robinia pseudoacacia* under different drought regimes. Means and SEs in brackets are shown. Bold digits indicate significant differences between drought treatments and control (*P* < 0.05). Growth variables were differences between the biomass obtained at plant mortality and that obtained at the beginning of the experiment.

	Fast drought		Slow drought	
	Control	Drought	Control	Drought
Basal diameter (mm)	4.51 (2.52)	−**2.04** (1.18)	5.93 (2.18)	−**0.37** (1.72)
Height (cm)	25.17 (4.07)	**1.33** (1.37)	41.67 (7.97)	**9.33** (1.75)
Main root (g)	15.95 (2.61)	16.56 (4.09)	21.99 (3.79)	**14.86** (3.04)
Coarse root (g)	11.82 (2.26)	**0.35** (1.84)	21.06 (1.98)	**1.16** (2.24)
Fine root (g)	8.79 (1.76)	−**6.97** (1.39)	14.28 (2.35)	−**9.94** (1.61)
Stem (g)	17.99 (7.15)	−**0.19** (1.18)	48.05 (8.21)	**1.16** (1.44)
Total biomass (g)	58.45 (10.06)	**10.26** (4.28)	114.29 (9.38)	**7.23** (3.68)
Survival time (days)		74.90 (2.31)		101.97 (4.14)

**Table 3.** Growth and survival time of *Platycladus orientalis* under different drought regimes. Means and standard errors in brackets are shown. Bold digits indicate significant differences between drought treatments and control (*P* < 0.05). Growth variables were differences between the biomass obtained at plant mortality and that obtained at the beginning of the experiment.

	Fast drought		Slow drought	
	Control	Drought	Control	Drought
Basal diameter (mm)	3.59 (2.11)	−**2.51** (0.77)	5.29 (1.76)	−**2.04** (1.33)
Height (cm)	18.33 (3.72)	**0.17** (1.47)	33.5 (5.21)	**0.83** (1.17)
Main root (g)	6.8 (2.39)	**14.55** (4.1)	9.83 (2.32)	13.1 (4.42)
Coarse root (g)	8.87 (2.54)	**1.31** (1.94)	14.02 (3.16)	**4.36** (2.36)
Fine root (g)	17 (4.46)	−**7.8** (1.4)	27.93 (1.93)	−**5.87** (2.54)
Stem (g)	36.27 (5.89)	**4.53** (1.81)	59.62 (13.97)	**31.47** (4.23)
Needle (g)	31.05 (8.09)	**3.54** (1.49)	41.95 (8.47)	**12.27** (0.87)
Total biomass (g)	99.99 (11.59)	**16.13** (5.43)	153.35 (20.61)	**55.33** (5.68)
Survival time (days)		75.43 (4.92)		132.03 (5.51)

### Growth change

For both species in FD and SD, all growth variables except for main root growth in FD *R. pseudoacacia* and FD and SD *P. orientalis* were significantly lower than those of the control (*P* < 0.05) (Tables 2 and 3). For *R. pseudoacacia*, the total biomass increment of SD plants was lower than that of FD plants, but the difference was not significant (*P* = 0.22). In contrast, for *P. orientalis*, the total biomass increment of SD plants was significantly higher than that of FD plants (*P* < 0.05).

### Water potential

**Figure 1.**
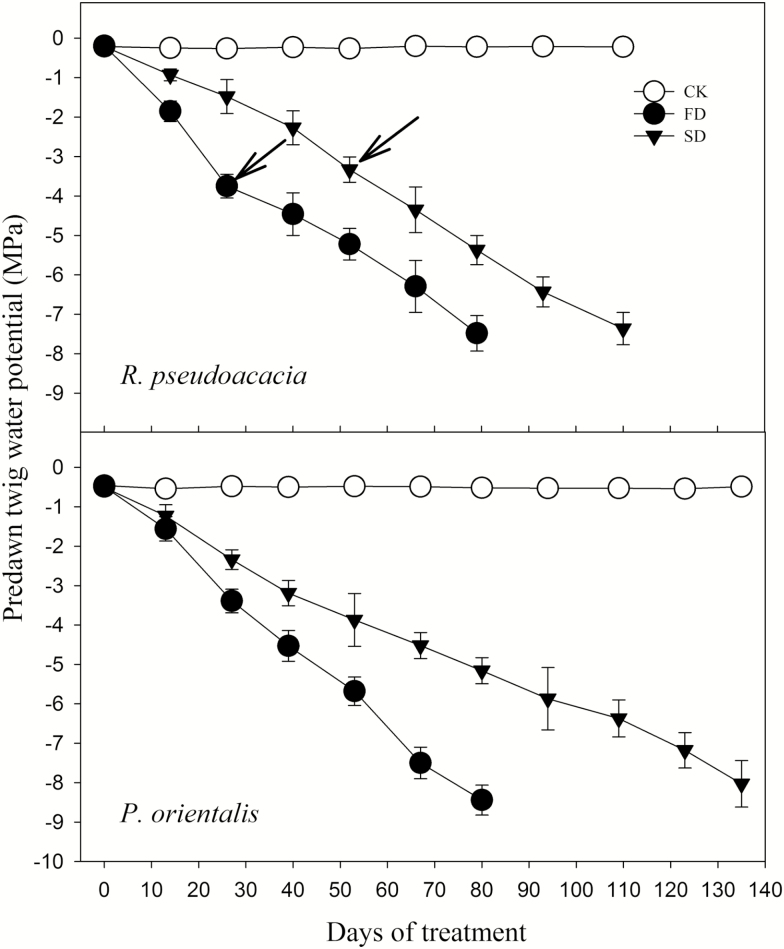
Change in predawn water potential during treatment. Arrows indicate time of complete defoliation. CK, FD and SD denote control, fast drought and slow drought, respectively.

The predawn water potential (Ψ_pd_) of both species declined with soil drying (Fig. 1). For each species, the Ψ_pd_ in the SD plants declined more slowly than that of the FD plants but both of them were nearly equal at mortality (~ −8 MPa for *R. pseudoacacia*, with no significant difference (*P* = 0.69), and <−8 MPa for *P. orientalis*, with no significant difference (*P* = 0.23)). For FD treatment, however, the *P. orientalis* Ψ_pd_ at mortality was significantly lower than that of *R. pseudoacacia* (*P* < 0.05), while under SD treatment there was no significant difference in the Ψ_pd_ between the two species (*P* = 0.07). There was significant interaction (*P* < 0.01) of drought intensity and duration on Ψ_pd_.

### PLC

**Figure 2.**
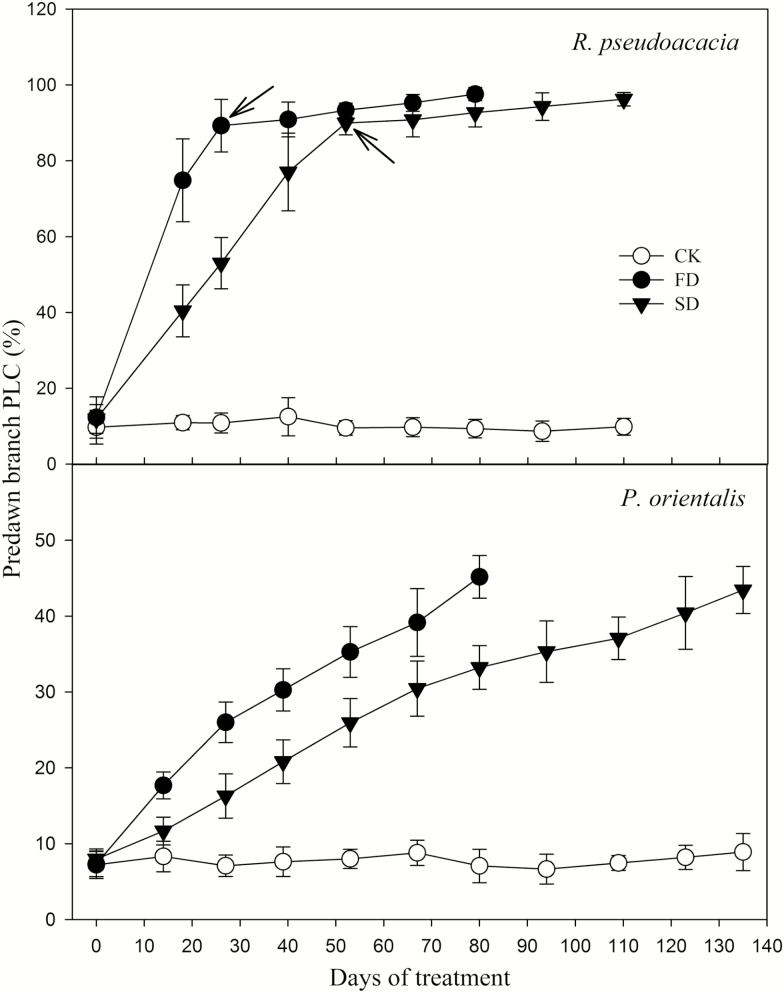
PLC (percentage loss of conductivity) changes during treatment. Arrows indicate time of complete defoliation. CK, FD and SD denote control, fast drought and slow drought, respectively.

For both species, drought intensity, duration and their interaction had significant effects on the predawn PLC (Fig. 2). The predawn PLC increased with soil drying rapidly in FD plants and slowly in SD plants. Nevertheless, they both reached the same maximum at the time of death (*P* = 0.30; Table 1). The PLC of *R. pseudoacacia* was >95 % at mortality. Upon complete defoliation, both FD and SD *R. pseudoacacia* had a PLC of ~90 % but it increased very slowly thereafter until death. For *P. orientalis*, however, the PLC of the dehydrated plants gradually reached a maximum of ~45 % at mortality, regardless of FD or SD treatments (*P* = 0.40). This level was attained faster in FD plants than SD plants (Fig. 2).

### Stomatal conductance and photosynthetic rates

**Figure 3.**
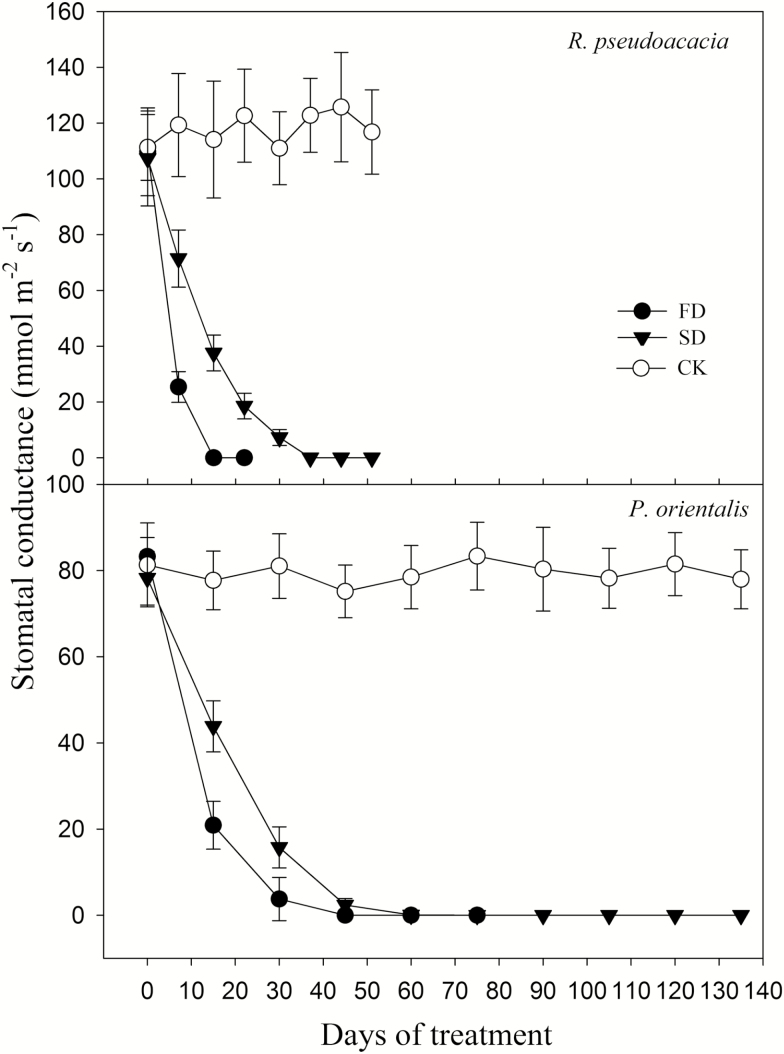
Change in stomatal conductance during drought. CK, FD and SD denote control, fast drought and slow drought, respectively.

**Figure 4.**
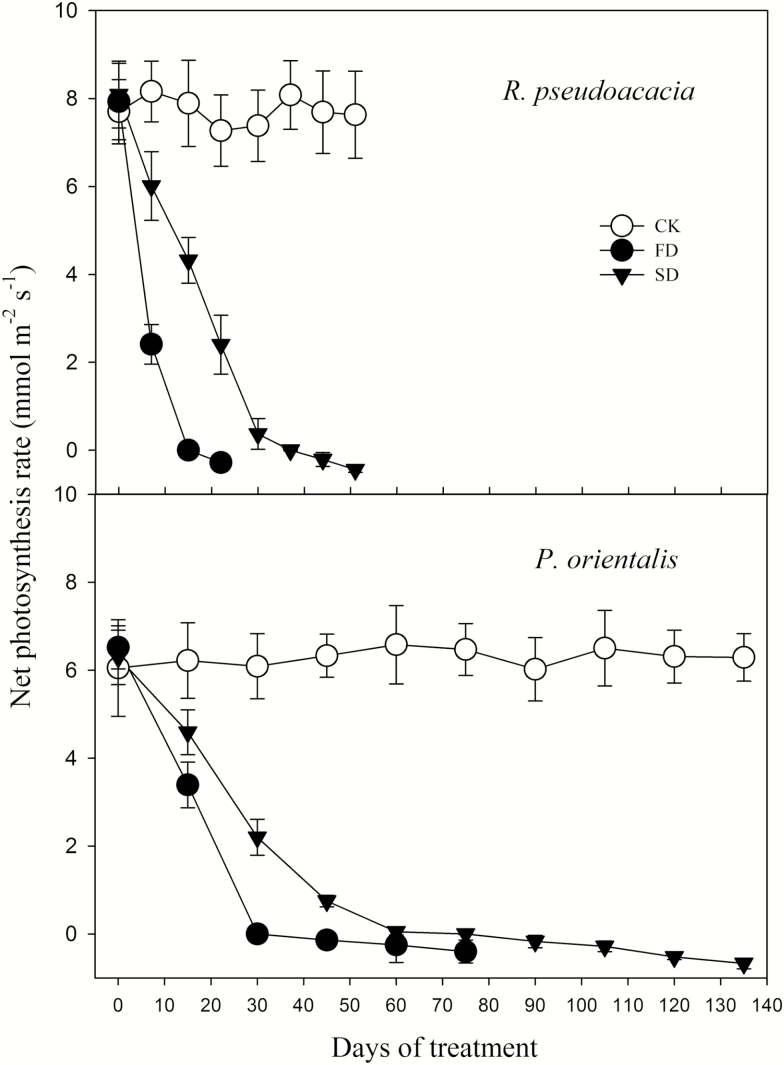
Change in net photosynthetic rate during drought. CK, FD and SD denote control, fast drought and slow drought, respectively.

The stomatal conductance in both species rapidly declined to ~0 during drought stress (Fig. 3). There were no significant differences in stomatal conductance at mortality between FD and SD plants (*P* > 0.05), although the stomatal conductance in FD plants decreased faster than that of SD plants. For both species, the net photosynthetic rate of all plants rapidly declined to <0 during drought (Fig. 4). For *R. pseudoacacia*, the net photosynthetic rate was <0 after 50 % defoliation (~15 days and ~37 days after treatment onset in FD and SD, respectively). The photosynthetic rate of *P. orientalis* was positive for ~1 month and >2 months for FD and SD, respectively. It decreased to <0 thereafter until plant death.

### Carbohydrate concentration changes

**Figure 5.**
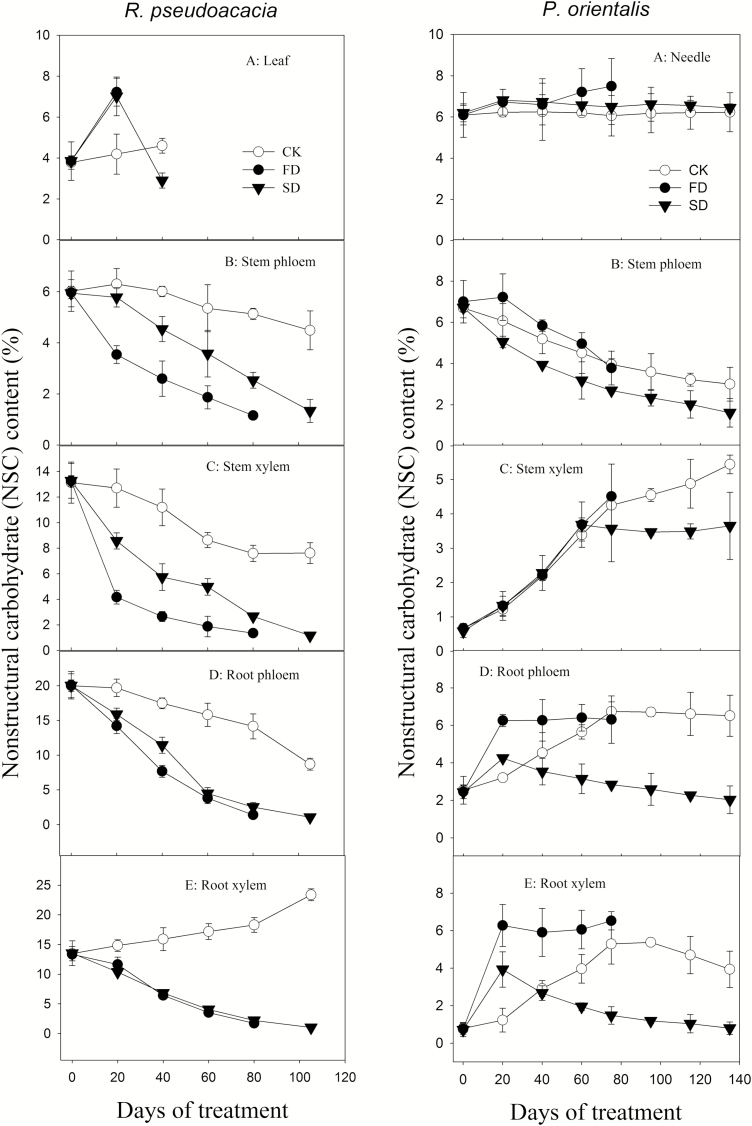
Change in NSC (non-structural carbohydrate) content during drought. A–E in the left panel are the NSC content of the leaves, stem phloem, stem xylem, root phloem and root xylem of *Robinia pseudoacacia*, respectively. A–E in the right panel are the NSC content of the needles, stem phloem, stem xylem, root phloem and root xylem of *Platycladus orientalis*, respectively.

During the experiment, the NSC concentrations in the stem and root phloem and xylem of both FD and SD *R. pseudoacacia* decreased significantly (*P* < 0.05) compared to the control (Table 4), declining to <2 % at mortality (Fig. 5, left panel B–E). The NSC in the stems of FD *R. pseudoacacia* declined more rapidly than that of the SD. The root NSC, however, decreased at a similar rate for both. The leaf NSC was 7.2 % in FD *R. pseudoacacia* before complete defoliation and significantly (*P* < 0.05) higher than that of the control (4.2 %). The NSC in SD *R. pseudoacacia* increased to 7 % in the first 20 days and then declined to 2.9 %, which was significantly (*P* < 0.05) lower than that of the control (4.6 %). At defoliation, the leaf NSC was still >2 % in both FD and SD *R. pseudoacacia* saplings. There were no significant differences in NSCs of all organs except for leaves between FD and SD plants (*P* > 0.05) (Table 1).

**Table 4.** The statistical significance (*P* value) of the effect of drought stress level, time and their interaction on studied parameters. Significant at α = 0.05. RP, *Robinia pseudoacacia*; PO, *Platycladus orientalis*; DF, degrees of freedom; NSC, non-structural carbohydrate; stem-p, stem phloem; stem-x, stem xylem; root-p, root phloem; root-x, root xylem.

Parameter	DF	*F* value	*P* value	Parameter	DF	*F* value	*P* value
Sugar of RP leaf				Sugar of PO leaf			
Stress	2	0.18	0.83	Stress	2	47.38	<0.01
Time	2	11.06	<0.01	Time	7	36.05	<0.01
Stress × Time	3	4.10	0.03	Stress × Time	11	2.28	0.03
Error	16			Error	42		
Starch of RP leaf				Starch of PO leaf			
Stress	2	4.58	0.03	Stress	2	19.65	<0.01
Time	2	23.53	<0.01	Time	7	29.06	<0.01
Stress × Time	3	7.95	<0.01	Stress × Time	11	0.95	0.51
Error	16			Error	42		
NSC of RP leaf				NSC of PO leaf			
Stress	2	3.85	0.04	Stress	2	2.92	0.07
Time	2	30.71	<0.01	Time	7	0.40	0.90
Stress × Time	3	13.31	<0.01	Stress × Time	11	0.36	0.96
Error	16			Error	42		
Sugar of RP stem-p				Sugar of PO stem-p			
Stress	2	28.48	<0.01	Stress	2	26.95	<0.01
Time	5	2.43	0.07	Time	7	22.90	<0.01
Stress × Time	9	5.13	<0.01	Stress × Time	11	1.74	0.10
Error	34			Error	42		
Starch of RP stem-p				Starch of PO stem-p			
Stress	2	50.12	<0.01	Stress	2	15.03	<0.01
Time	5	52.51	<0.01	Time	7	17.55	<0.01
Stress × Time	9	2.76	0.02	Stress × Time	11	0.78	0.66
Error	34			Error	42		
NSC of RP stem-p				NSC of PO stem-p			
Stress	2	109.43	<0.01	Stress	2	25.52	<0.01
Time	5	50.82	<0.01	Time	7	38.64	<0.01
Stress × Time	9	7.72	<0.01	Stress × Time	11	1.20	0.32
Error	34			Error	42		
Sugar of RP stem-x				Sugar of PO stem-x			
Stress	2	41.26	<0.01	Stress	2	9.17	<0.01
Time	5	18.13	<0.01	Time	7	26.86	<0.01
Stress × Time	9	4.12	<0.01	Stress × Time	11	2.90	<0.01
Error	34			Error	42		
Starch of RP stem-x				Starch of PO stem-x			
Stress	2	210.65	<0.01	Stress	2	4.37	0.02
Time	5	170.40	<0.01	Time	7	25.93	<0.01
Stress × Time	9	13.24	<0.01	Stress × Time	11	0.41	0.95
Error	34			Error	42		
NSC of RP stem-x				NSC of PO stem-x			
Stress	2	206.79	<0.01	Stress	2	9.53	<0.01
Time	5	147.56	<0.01	Time	7	78.96	<0.01
Stress × Time	9	13.14	<0.01	Stress × Time	11	2.87	<0.01
Error	34			Error	42		
Sugar of RP root-p				Sugar of PO root-p			
Stress	2	87.42	<0.01	Stress	2	33.65	<0.01
Time	5	13.90	<0.01	Time	7	5.03	<0.01
Stress × Time	9	16.66	<0.01	Stress × Time	11	2.98	<0.01
Error	34			Error	42		
Starch of RP root-p				Starch of PO root-p			
Stress	2	174.15	<0.01	Stress	2	68.92	<0.01
Time	5	243.34	<0.01	Time	7	17.09	<0.01
Stress × Time	9	13.79	<0.01	Stress × Time	11	12.62	<0.01
Error	34			Error	42		
NSC of RP root-p				NSC of PO root-p			
Stress	2	236.45	<0.01	Stress	2	95.05	<0.01
Time	5	247.38	<0.01	Time	7	13.90	<0.01
Stress × Time	9	18.58	<0.01	Stress × Time	11	10.04	<0.01
Error	34			Error	42		
Sugar of RP root-x				Sugar of PO root-x			
Stress	2	82.36	<0.01	Stress	2	58.41	<0.01
Time	5	9.92	<0.01	Time	7	10.93	<0.01
Stress × Time	9	22.48	<0.01	Stress × Time	11	6.25	<0.01
Error	34			Error	42		
Starch of RP root-x				Starch of PO root-x			
Stress	2	430.56	<0.01	Stress	2	82.14	<0.01
Time	5	34.65	<0.01	Time	7	23.17	<0.01
Stress × Time	9	38.10	<0.01	Stress × Time	11	14.46	<0.01
Error	34			Error	42		
NSC of RP root-x				NSC of PO root-x			
Stress	2	578.71	<0.01	Stress	2	108.60	<0.01
Time	5	44.77	<0.01	Time	7	23.98	<0.01
Stress × Time	9	61.98	<0.01	Stress × Time	11	14.47	<0.01
Error	34			Error	42		

The NSC in the stems of FD-treated *P. orientalis* did not significantly (*P >* 0.05) differ from that of the control until mortality. In contrast, the root NSC of FD *P. orientalis* rapidly increased in the first 20 days and then dropped off to control level (~6 %) at mortality (Fig. 5, right panel A). There were no significant differences in needle NSC (*P* > 0.05) among the three treatments (Table 4). The stem and root NSC levels in SD *P. orientalis* were significantly lower (*P* < 0.05) than those of the control at mortality. The root NSC concentrations in SD *P. orientalis* increased in the first 20 days and then declined to 0.8 and 2.0 % at mortality for the xylem and phloem, respectively. In both SD and FD plants, the NSC concentrations were >2 % for the stems but ≤2 % for the roots. The SD treatment consumed more NSC than the FDs. The NSCs in roots and stem phloem of SD plants at mortality were significantly lower than those of FD plants (Table 1). A comparison between species showed that *P. orientalis* retained more NSCs at mortality than *R. pseudoacacia*, especially for the FD plants.

**Figure 6.**
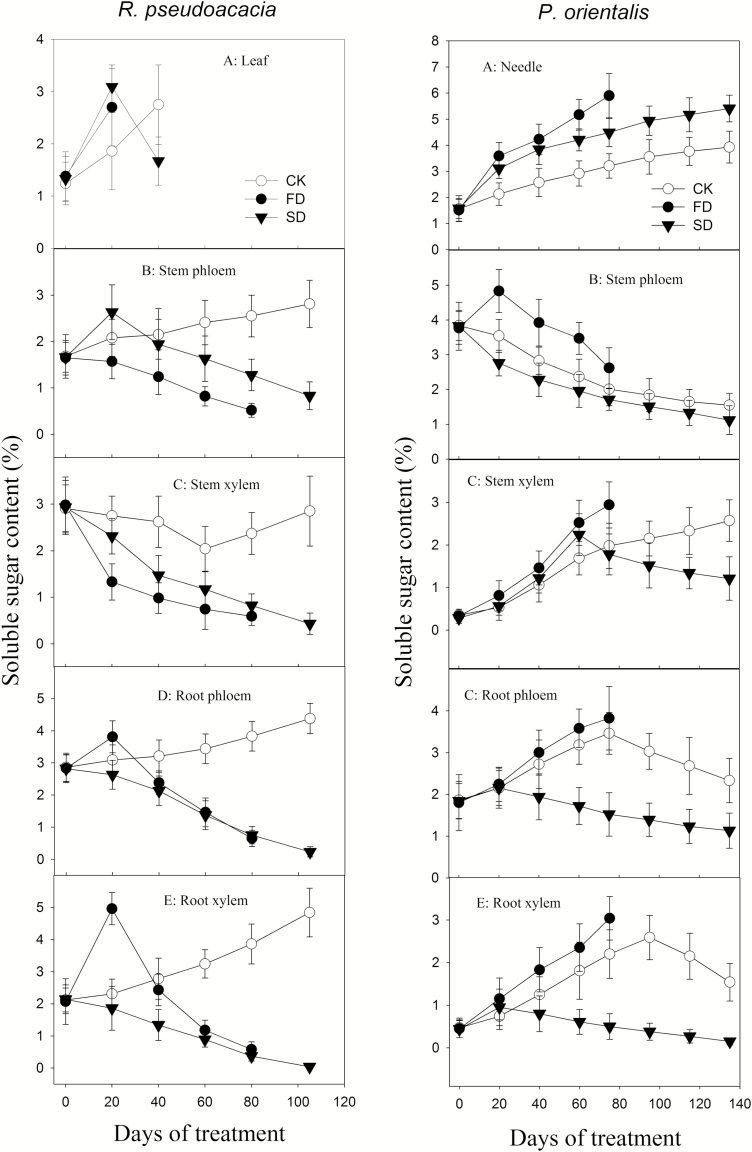
Change in SS (soluble sugar) content during drought. A–E in the left panel are the SS content of the leaves, stem phloem, stem xylem, root phloem and root xylem of *Robinia pseudoacacia*, respectively. A–E in the right panel are the SS content of the needles, stem phloem, stem xylem, root phloem and root xylem of *Platycladus orientalis*, respectively.

Except for the stem xylem, the SS content in the control *R. pseudoacacia* increased. In contrast, the root and stem SS decreased to <1 % at mortality in the drought-treated *R. pseudoacacia* (Fig. 6, left panel A–E). Nevertheless, in *R. pseudoacacia*, the SS in the SD stem phloem and the FD root phloem and xylem increased early in the treatment (Fig. 6, left panel B, D and E). Only FD-treated *R. pseudoacacia* plants had significantly higher leaf SS content than the control (*P* < 0.05). For FD *P. orientalis*, the SS content in all organs and tissues except the stem phloem increased and were somewhat higher than those of the control at mortality (*P* < 0.05 for needle; *P* > 0.05 for stem xylem, root phloem and root xylem). The stem phloem SS declined in all treatments. In FD plants, however, it increased at the early stage and was slightly higher (*P* > 0.05) than that of the control (Fig. 6, right panel A–E). The SD *P. orientalis* had relatively lower SS than the control at death (*P* < 0.05 for stem xylem, root phloem and root xylem; *P* > 0.05 for stem phloem) except for the needles. The SS of the stem xylem, root phloem and root xylem in SD *P. orientalis* briefly increased at the early stage (Fig. 6, right panel A–E). The overall needle SS content was significantly higher (*P* < 0.05) than that of the control.

**Figure 7.**
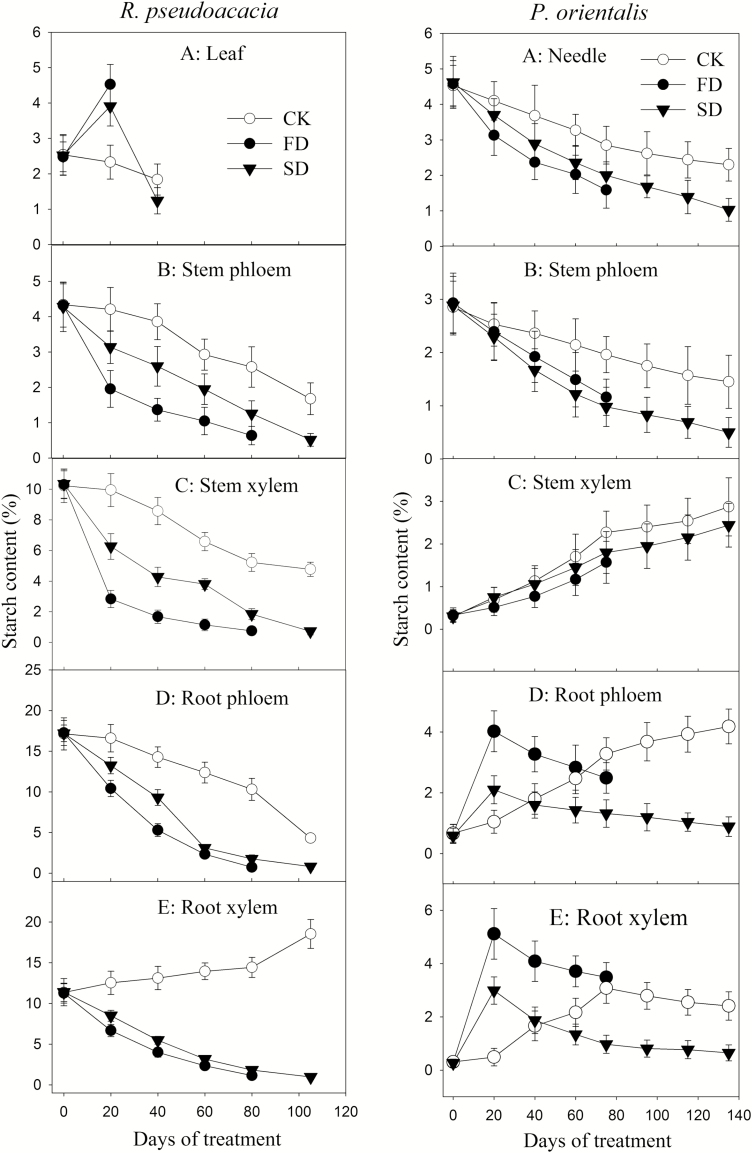
Change in St (starch) content during drought. A–E in the left panel are the St content of the leaves, stem phloem, stem xylem, root phloem and root xylem of *Robinia pseudoacacia*, respectively. A–E the in right panel are the St content of needles, stem phloem, stem xylem, root phloem and root xylem of *Platycladus orientalis*, respectively.

The St content in both FD and SD *R. pseudoacacia* plants rapidly decreased and was significantly lower (~0; *P* < 0.05) at mortality than that of the control except for the leaf St of FD plants, which was significantly higher (*P* < 0.05) than that of the control at defoliation (~20 days after the start of treatment) (Fig. 7, left panel A–E). For *P. orientalis*, St in the aerial shoot in drought-treated plants was somewhat lower than that of the control but changed in almost the same way. The St content in the root phloem and xylem of drought-treated *P. orientalis* increased rapidly at the early stage and then declined (Fig. 7, right panel A–E). In *P. orientalis roots*, St increased in FD more than it did in SD but declined to the control level at death. Starch in SD *P. orientalis* roots was significantly lower at mortality than that of the control. The needle and stem phloem St in both FD and SD *P. orientalis* were significantly (*P* < 0.05) lower than those of the control.

## Discussion

Drought reduced plant growth and photosynthetic rate, promoted PLC and altered carbon metabolism. At the time of death, there was no effect of drought type (SD vs. FD) on PLC in either species, but there were significant differences in PLC between species (95 % in *R. pseudoacacia*; 45 % in *P. orientalis*). Carbon levels significantly differed between species and drought conditions. All drought-stressed saplings experienced hydraulic failure, but carbon depletion varied among different plants.

### The role of hydraulic failure

At mortality, the PLC of all drought-treated *R. pseudoacacia* saplings was >95 % and the Ψ_pd_ ~ −8 MPa. All drought-treated *P. orientalis* saplings had PLC **~**45 % and Ψ_pd_ < −8 MPa. The PLC and Ψ_pd_ for each treatment differed significantly between the two species. Nevertheless, the results are consistent with those of Choat (2013), who reported that gymnosperm species died at ~P_50_, whereas angiosperms died at ~P_88_. All drought-stressed saplings underwent irreversible embolism, which may cause plant death by tissue desiccation and hydraulic failure (Brodribb and Cochard 2009; Urli *et al.* 2013). The latter depends on plant water status. When the water potential threshold is reached, hydraulic failure may occur. As water stress increases, the water potential declines and the embolism accumulates. Therefore, the critical water potential threshold may be attained regardless of the duration of the drought. As observed in this study, both FD and SD saplings may suffer hydraulic failure. This result contradicts our expectation.

Gymnosperms often have wider hydraulic safety margins than angiosperms (Choat *et al.* 2012) but both have roughly the same mortality risk under drought (Anderegg *et al.* 2016). Due to low quantities of NSC and xylem parenchyma in their stems, both of which may be required for embolism repair (Johnson *et al.* 2012; Urli *et al.* 2013), gymnosperms may not recover from 50 % conductivity loss (Ogasa *et al.* 2013; Anderegg *et al.* 2016). Once it occurs, a gymnosperm may undergo catastrophic hydraulic failure (Meinzer and McCulloh 2013; Anderegg *et al.* 2016). There are differences in the critical PLC threshold between angiosperm and gymnosperm (88 vs. 50 %) and between *R. pseudoacacia* and *P. orientalis* (95 vs. 45 %). The water potential corresponding to 45 % conductivity loss in *P. orientalis* was between −9 and −8 MPa. This value was significantly lower than that corresponding to 95 % conductivity loss in *R. pseudoacacia* (~ −8 MPa). The PLC in both FD- and SD-treated *R. pseudoacacia* rapidly increased to ~90 % before complete defoliation. Nevertheless, the trees lived for around 46 days after leaf abscission. Since plants may survive for months with zero hydraulic conductivity (McDowell, 2011), a different mechanism is involved in plant death.

### The role of carbon depletion

*Robinia pseudoacacia* rapidly responded to drought by shedding its leaves. Defoliation is a common drought response strategy (Galvez *et al.* 2011; Ryan 2011). Easily defoliated species may readily regenerate leaves or root suckers when irrigated (Lu *et al.* 2010; Galvez *et al.* 2011) (‘recovery’ strategy; Michell *et al.* 2013). Defoliation minimizes water loss and reduces embolism accumulation during drought. These strategies help plants contend with brief or intense drought. Nevertheless, they invariably suffer from carbon depletion due to the ongoing carbon-consuming physiological activities during near-zero photosynthesis (Poyatos *et al.* 2013; Salmon *et al.* 2015). Both FD- and SD-treated *R. pseudoacacia* nearly depleted their NSC at death and there was no treatment effect on carbon level. Carbon depletion may be correlated with hydraulic dysfunction (Sevanto *et al.* 2014; Aguadé *et al.* 2015; Gaylord *et al.* 2015; Serrana *et al.* 2015). Non-structural carbohydrate plays a key role in osmotic adjustment, embolism refilling and root system growth (Gieger and Thomas 2002; Sala *et al.* 2012). Severe fine root biomass reduction may restrict water uptake and impair water relations (Gieger and Thomas, 2002). Carbon depletion can exacerbate hydraulic dysfunction. Carbon depletion and hydraulic failure coincided in *R. pseudoacacia.* Contrary to our hypothesis, carbon depletion could occur under either drought condition.

Unlike *R. pseudoacacia*, *P. orientalis* did not defoliate in response to water stress. Its needles did not wither, fall or die until tree mortality (‘waiting it out’ strategy; Michell *et al.* 2013). It had longer periods of >0 photosynthesis than *R. pseudoacacia* (~1 month and ~2 months for FD and SD, respectively). The result is consistent with previous report (Brodribb *et al.* 2014), in which *P. orientalis* along other Cupressaceae species maintains relative prolong stomatal opening and photosynthetic activity during drought. The NSC at mortality varied between FD- and SD-treated *P. orientalis*. Fast drought saplings had the same NSC as the control plants, whereas the NSC of the SD saplings was significantly less than those of the control trees. Slow drought significantly reduced NSC in both species, whereas FD had different effects on NSC levels between the two species. Slow drought *P. orientalis* saplings can utilize their carbon stores; however, they can still succumb to lethal embolism. In terms of hydraulic aspect, *P. orientalis* saplings gradually increased their PLC during drought until catastrophic embolism occurred. Both FD and SD saplings, then, experienced hydraulic failure regardless of carbon reserves.

## Conclusion

This study explored the effects of hydraulic failure and carbon starvation on sapling mortality under various drought conditions. Under FD treatment, *P. orientalis* (a gymnosperm) only suffered hydraulic failure, whereas *R. pseudoacacia* (an angiosperm) experienced both hydraulic failure and carbon depletion. Unexpectedly, all water-stressed plants experienced hydraulic failure, and even slow soil drying did not prevent it. A combination of hydraulic failure and carbon depletion contributed to the death of *R. pseudoacacia*, whereas hydraulic failure was the main mortality factor in *P. orientalis*. Drought characteristics and tree attributes influence the physiological pathways leading to plant death. The observations of the present study will aid in understanding the mechanisms of drought-induced tree mortality and predicting forest responses to climate change.

## Sources of Funding

This study has been supported by grants from the National Natural Science Foundation of China (Nos. 31270648 and 31290223).

## Contributions by the Authors

Y.D. performed the study and analysed data; L.W. conducted part of experimental measurements in field; X.W. conceived the paper and coordinated the research project; all authors contributed in writing the manuscript.

## Conflict of Interest

None declared.

## Supporting Information

The following additional information is available in the online version of this article—

**Table S1**. Dimension of seedlings before treatment.

## Supplementary Material

Supporting Information
