# Supplementary material for: Relative contributions of hydraulic dysfunction and carbohydrate depletion during tree mortality caused by drought
Source: AoB Plants. 2017 Dec 8;10(1):plx069. doi: 10.1093/aobpla/plx069 (PMC5774510; doi:10.1093/aobpla/plx069)
Supplement: Supporting Information [file plx069_suppl_supporting_information.docx]

Table S1 Dimension of seedlings before treatments

|  | Fast drought | | Slow drought | |
| --- | --- | --- | --- | --- |
|  | Control | Drought | Control | Drought |
| *R. pseudoacacia* |  |  |  |  |
| Basal diameter (mm) | 21.2(3.3) | 21.7(3.8) | 20.9(2.9) | 21.2(1.5) |
| Height (cm) | 91.3(4.7) | 88.7(4.1) | 90.3(3.7) | 90.2(4.3) |
| *P. orientalis* |  |  |  |  |
| Basal diameter (mm) | 18.7(2.9) | 19.4(2.1) | 18.7(2.9) | 21.5(3.4) |
| Height (cm) | 108.7(14.0) | 113.5(9.9) | 108.7(14.0) | 112.7(13.7) |

Means and standard errors in brackets are shown. There were no significant differences in the variables between the corresponding control and treatment pairs.
